# Threshold-Free Population Analysis Identifies Larger DRG Neurons to Respond Stronger to NGF Stimulation

**DOI:** 10.1371/journal.pone.0034257

**Published:** 2012-03-27

**Authors:** Christine Andres, Jan Hasenauer, Frank Allgower, Tim Hucho

**Affiliations:** 1 Max Planck Institute for Molecular Genetics, Berlin, Germany; 2 Institute of Chemistry and Biochemistry, Freie Universität Berlin, Berlin, Germany; 3 Institute for Systems Theory and Automatic Control, University of Stuttgart, Stuttgart, Germany; Indiana University School of Medicine, United States of America

## Abstract

Sensory neurons in dorsal root ganglia (DRG) are highly heterogeneous in terms of cell size, protein expression, and signaling activity. To analyze their heterogeneity, threshold-based methods are commonly used, which often yield highly variable results due to the subjectivity of the individual investigator. In this work, we introduce a threshold-free analysis approach for sparse and highly heterogeneous datasets obtained from cultures of sensory neurons. This approach is based on population estimates and completely free of investigator-set parameters. With a quantitative automated microscope we measured the signaling state of single DRG neurons by immunofluorescently labeling phosphorylated, i.e., activated Erk1/2. The population density of sensory neurons with and without pain-sensitizing nerve growth factor (NGF) treatment was estimated using a kernel density estimator (KDE). By subtraction of both densities and integration of the positive part, a robust estimate for the size of the responsive subpopulations was obtained. To assure sufficiently large datasets, we determined the number of cells required for reliable estimates using a bootstrapping approach. The proposed methods were employed to analyze response kinetics and response amplitude of DRG neurons after NGF stimulation. We thereby determined the portion of NGF responsive cells on a true population basis. The analysis of the dose dependent NGF response unraveled a biphasic behavior, while the study of its time dependence showed a rapid response, which approached a steady state after less than five minutes. Analyzing two parameter correlations, we found that not only the number of responsive small-sized neurons exceeds the number of responsive large-sized neurons—which is commonly reported and could be explained by the excess of small-sized cells—but also the probability that small-sized cells respond to NGF is higher. In contrast, medium-sized and large-sized neurons showed a larger response amplitude in their mean Erk1/2 activity.

## Introduction

Tissues, primary cells, and even clonal cells are heterogeneous, e.g., in terms of morphology, protein expression, metabolite concentrations, and signaling status [1]–[9]. This heterogeneity is often crucial for processes such as differential stimulus sensing [5] and robust decision-making [6]–[9]. The analysis of population heterogeneity and the underlying subpopulations allows insight into the cellular functionality. But the analysis of heterogeneous populations is challenging. Of particular importance for the comparability of results between different research groups are methods to detect and characterize subgroups which do not rely on often ill-defined investigator-dependent parameters for measurement and classification. In addition, most available analysis tools require that an “average cell” exists, or at least assume normally distributed subpopulation properties [10]. As this is not true for most heterogeneous biological populations more sophisticated analysis tools are required.

One particular problem which requires understanding of cellular heterogeneity, is pain. Pain evoking stimuli are detected by peripheral sensory neurons – so called DRG neurons –, transmitted along the neuron, via the dorsal root ganglion, to the spinal cord. There, secondary neurons are activated to produce the experience of pain in the brain. DRG neurons detect diverse environmental stimuli such as temperature, touch, or chemicals. As individual DRG neurons often detect only a subset of these stimuli, they are functionally highly heterogeneous. They differ in stimulus responsiveness but also, for example, in cell size, protein content, and innervation area [5].

Diverse classification criteria have been applied for determining the highly overlapping subgroups of DRG neurons, such as anatomical properties [11], electrophysiological firing patterns [12], and/or protein expression [13]. But, clinical relevant pain is focussed on a further cause of heterogeneity, sensitization. Mediators, for example, present in inflamed tissue, initiate the sensitization of signaling cascades which often results in stronger and prolonged activation of sensory neurons to pain eliciting stimuli. Furthermore, stimuli which are normally not perceived as painful become strongly painful [14]. Recent studies showed, that sensitization signaling can be investigated on a single cell level by following the degree of signaling component activation and their kinetics [15], [16].

One challenge of investigations of heterogeneity such as sensitization signaling is the necessity to measure quantitative data of gradual signals in single cells and not just to assume a binary marker-positive versus marker-negative signal. To accomplish this, we introduced recently a quantitative automated microscopy (QuAM) approach for the study of sensitization signaling such as the MAP-kinase pathway in DRG neurons [16]. The advantage of QuAM compared to common methods for monitoring pathway activation, e.g., Western blotting, is its single cell resolution. Furthermore, QuAM allows for the analysis of hundreds to thousands of single cells providing decently large datasets of adherent cells, which is the prerequisite for the quantitative assessment of populations in heterogeneous cell systems.

**Figure 1:**
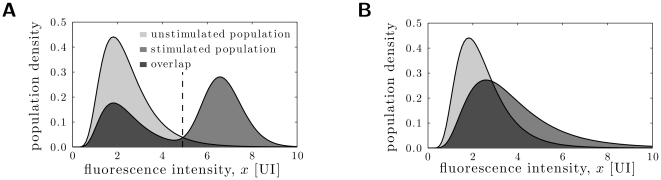
The suitability of threshold methods to heterogeneous cell populations depends on the response magnitude of individual cells. **A** Thresholding methods are appropriate tools to distinguish responsive and unresponsive subgroups in stimulated cell cultures, if responding cells show a much higher fluorescence intensity than non-responding cells. **B** Thresholding methods do not provide quantitative information about the size of responsive and unresponsive subgroups, if responding and non-responding cell populations overlap to a large extent. In such cases an actual biological threshold does not exist and every threshold-based method will result in a large numbers of false positive and false negative cells. (This is a schematic and does not show actual measurement data. The abbreviation [UI] denotes the unit of intensity and thus the unit of relative fluorescence.).

So far, the analysis of subgroups in DRG neurons is almost exclusively based on thresholding methods. Common thresholding methods use a subjective investigator-chosen cutoff-criterion for one cell property, e.g., fluorescence intensity of an immunofluorescently labeled protein, and quantify the portion of cells below and above the threshold. Thereby, the cells are classified as negative or positive with respect to the observed cell property. Differences in the subjective threshold results in large variation of reported population sizes, for example between 30–100% for TRPV1-positive neurons [17], [18]. But also beyond the problems inherent to subjective parameter setting, the quality of thresholding-based results depends on the degree of heterogeneity and the dynamic range of the investigated effect. For nearly non-overlapping subgroups (Figure 1A) it is easy to find an appropriate threshold, while for highly overlapping subgroups (Figure 1B) with low dynamic range, such as found in the heterogeneous populations of DRG neurons, the selection of an appropriate threshold is problematic [19]. In such cases, thresholding methods are prone to yield false positive and/or false negative results.

To overcome the drawbacks of thresholding, various threshold-free histogram-based methods were introduced [19]–[21]. These methods have so far not been applied to analyze data derived from DRG neurons, probably, because the required number of single cell measurements are in the tens of thousands [19]. Such large datasets are commonly not available when studying primary neurons as only few tens of thousands sensory neurons exist per animal. One reason for the large amount of required single cell measurements is the loss of information caused by binning [22]. In addition, suboptimal binning can result in severe misinterpretations [22].

In the following, we introduce a novel tool – called *KDE subtraction method* – to quantify the subpopulation size in highly heterogeneous cell systems of relatively small cell numbers by exploiting kernel density estimation (KDE). The KDE subtraction method is like histogram-based approaches threshold-free and determines the percentage of responsive cells, irrespective of the response of the single cells. Therefore, the population density before and after the stimulus is analyzed, allowing for the assessment of the population change and of quantitative properties of the identified subpopulation. The number of cells required to obtain reliable estimates is thereby determined using bootstrapping, showing that our approach can also be applied to limited datasets containing hundreds of measured cells.

The method is employed to analyze the heterogeneous signaling state at baseline, as well as after treatment with the potent sensitizing substance, NGF, by measuring Erk1/2 phosphorylation. Thereby, we quantify for the first time the dependencies of responses on NGF dose and kinetics on a true population basis, and investigate novel functional aspects, such as the size-dependence of the responsiveness and its response amplitude.

## Materials and Methods

### Chemicals and drugs

BSA, L-glutamine, poly L-ornithine hydrochloride, DMSO, paraformaldehyde, Triton X-100 and glutamate were purchased from Sigma (Taufkirchen, Germany), collagenase P from Roche (Mannheim, Germany), trypsin from Worthington Biochemical Corporation (Freehold, NJ, USA), Neurobasal A (without phenol red), B27 supplement, laminin, minimum essential medium with glutamax were purchased from Invitrogen (Germany, UK), DMEM, trypsin and EDTA from Clonetics (Cambrex, US) and normal donkey serum from Dianova (Hamburg, Germany). mNGF was purchased from Alomone (Jerusalem, Israel).

### Antibodies

Anti-PGP 9.5 was purchased from MorphoSys AG (Martinsried/Planegg, Germany; final concentration 1∶1000). Anti-phospho-Erk (Thr-202/Tyr-204) was purchased from New England Biolabs (Frankfurt am Main, Germany; final concentration 1∶200). Alexa-594-labeled chicken anti-rabbit IgG and Alexa-488 chicken anti-mouse IgG were purchased from Molecular Probes Invitrogen (Karlsruhe, Germany; final concentration 1∶1000).

### Animals

Male Sprague Dawley rats were purchased from Harlan (Rossdorf, Germany). Our institution is licensed to house and work with these animals by the responsible authority (LaGeSo, Berlin, license ZH120). For tissue collection, rats were euthanized by 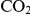 inhalation. This procedure was reported to and approved by the LAGeSo, Berlin (T0370/05).

### DRG-cultures

Cultures of dissociated DRG were prepared from male Sprague Dawley rats as described previously [15]. The rates were euthanized by 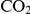 intoxication and L1–L6 DRGs were removed, desheathed, pooled and incubated with collagenase (final concentration (f.c.) 0.125%; 1 h, 37°C). The neurons were dissociated by trypsin digestion (f.c. 1176 u, 8 min, 37°C) and a trituration with a fire-polished Pasteur pipette. Axon stumps and dead cells were removed by centrifugation (5 min, 100 g). Viable cells were resuspended in 12 ml of NeurobasalA/B27 medium, plated 0.5 ml/culture onto polyornithine/laminin-precoated glass coverslips (12 mm diameter), and incubated overnight in 24 well plates at 37 in 5% 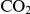.

### Cell stimulation

After incubation for 15–20 h, cells were stimulated with the growth factor NGF. To ensure homogeneous mixture of the stimulants, a volume of 250 µl out of the 500 µl culture medium was removed from the culture well, mixed thoroughly with the stimulant, and added back to the same culture. Negative controls were treated alike but without the addition of any reagent. To reduce mechanical cell stress the stimulus was added very slowly (250 µl in 6 s) using an automatic pipette (Multipette® pro from Eppendorf). After treatment, the cells were washed once with phosphate-buffered saline (PBS) and fixed with paraformaldehyde (4%, 10 min) at room temperature (RT).

### Immunocytochemistry

Paraformaldehyde-fixed cells were permeabilized with 0.1% Triton X-100 (10 min, RT), followed by three washes with PBS (5 min, RT). After blockage of nonspecific binding sites (5% bovine serum albumin (BSA) and 10% normal donkey serum in PBS; 1 h, RT), the cultures were probed with primary antibodies against target proteins (antibody concentrations against target proteins as indicated in the Section *Antibodies*) in 1% BSA in PBS (1 h, RT), washed three times (1% BSA in PBS; 5 min, RT), and incubated with secondary antibodies (1 h, RT). After three final washes (PBS; 5 min, RT), the cultures were mounted with Fluoromount-G (Southern Biotechï¿½ Biozol) containing DAPI (0.5 µg/ml).

### Quantitative automated microscopy (QuAM)

Cells were evaluated with a Zeiss Axioplan 2 microscope controlled by the software Metacyte (Metasystems). Images of 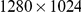 pixels were taken using a 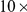 objective. The exposure time was defined automatically such that maximal 1000 pixel/100 µm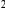 were saturated, but was maximal 0.96 s. For automatic neuron recognition the following parameters were defined: size (150–1500 µm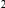), form (aspect ratio = 2; concavity depth = 0.25), contrast (object threshold 30%). The integrative pixel intensity of each selected neuron was normalized against the respective neuron area and exposure time. For cell identification the neuron specific PGP 9.5 immunostaining was used as independent selection marker. Fluorescence intensities derived from phospho-Erk1/2 antibody were quantified on independent color channels.

Employing this procedure, we obtained population data 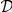, which were the collection of Erk1/2 phosphorylation amounts 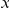 in hundreds of individual cells (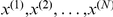),
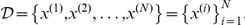
The superscript 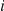 specified the individual cells within the population and 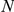 is the number or measured single cells under the considered condition.

### Computation of population density from QuAM derived data

Within a population, the 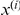
's are distributed according to the population density function 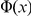. The variable 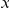 denotes in our study the level of phospho-Erk1/2 (pErk1/2) fluorescence (if not mentioned otherwise), which is always non-negative. Given 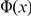 the probability of observing a single cell in the population with a level of fluorescence 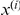 which is contained in the interval 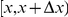 is
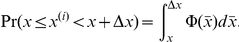
Furthermore, as 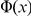 is a probability density, 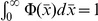 holds. For a more detailed introduction to probability densities we refer to [22]–[24]. The change of the population density 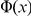 contains all available informations about the response of the cell population. Therefore, our analysis will focus on 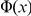.

**Figure 2:**
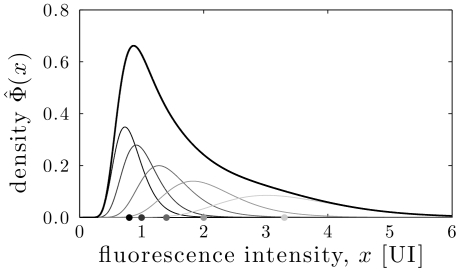
Illustration of kernel density estimation. The kernel density estimate 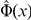 (thick line) of 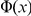 for the measured single-cell fluorescence intensities 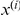 (gray dots) is constructed from the associated kernels 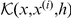 (gray lines).

Unfortunately, 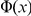 cannot be measured directly but 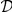, which is a sample from 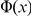. Given 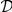, an approximation 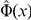 of 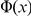 can be determined by kernel density estimation. Kernel density estimators are non-parametric methods to estimate probability density from sampled data [22]. They are widely used and can be thought of as placing probability “bumps” at each single data point 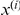, as illustrated in Figure 2. These “bumps” are the kernel functions 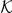 which are themselves probability densities,
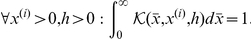
In this work, a log-normal distribution is used as kernels given by
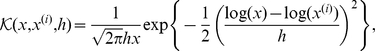
with kernel bandwidth 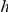 [22]. We chose log-normal kernels instead of the common Gaussian kernels, as log-normal kernels preserve the positivity of 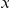. Note that here only the equations for the one-dimensional case are provided. The extension towards higher dimensions is straight forward and can be found in [22].

Given the kernel 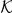 an estimator of the probability density 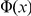 for using the sample 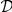 is
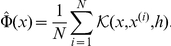
The selection of the smoothing parameter 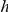 is crucial and depends strongly on 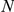 and the data 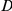. In this work 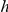 is chosen according to Silverman's rule of thumb [22].

### Estimation of the size of the responsive subpopulation

Employing the kernel density estimator induced above, we compute an estimate for the percentage of responsive cells based on two datasets:

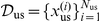
 … dataset of unstimulated (us) cells, and

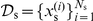
 … dataset of stimulated (s) cells.

**Figure 3:**
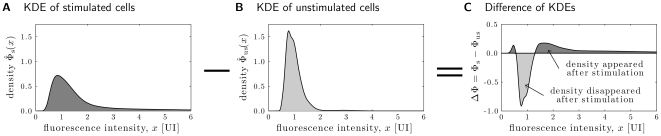
Procedure of KDE subtraction method. At first, the kernel density estimates of the datasets for stimulated cells **A** and unstimulated cells **B** are computed. Given these probability densities the difference density **C** is calculated. Integration over the positive part of this density yields the size of the responsive subpopulation 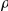 (for illustration purposes we chose a real dataset obtained by stimulation of DRG neurons for 1 hour with 1 nM NGF). The estimated size of the responsive subgroup is 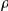 = 41%.

To estimate the percentage of responsive cells 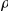, in a first step the kernel density estimates of the corresponding densities 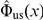 and 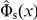 are determined using the data. The difference of 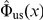 and 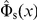 provides information about the change of the density due to the stimulus. Additionally, the size of the responsive subpopulation can be calculated by integration over the positive part of the density difference (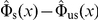):
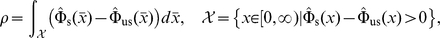
as depicted in Figure 3. Note that integration over the absolute value of the negative part of the difference density yields the same result. The change of the density due to the stimulus directly provides information about the population dynamics and 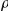 is an estimate for the portion of responsive cells. If the number of cells 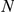 increases the approximations of the densities, 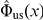 and 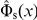, and the estimate of the subpopulation size 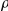 improve.

Note that the estimate 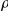 determined using the proposed KDE subtraction method may underestimates the true size of the responsive subgroup as it cannot be distinguished between: (1) the phosphorylation signal in one cell increases from 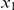 to 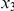; and (2) the phosphorylation signal in a cell increases from 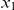 to 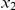, while the protein concentration in another cell increases from 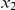 to 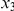. Both cases will yield the same final probability density 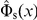 but in (2) twice as many cells responded as in (1). Thus, the proposed scheme suffers in this respect the same disadvantage as the (modified) histogram subtraction method [19]. The distinction of these two cases would require time-lapse single cell data of large population, which are not available for many systems. Alternatively, further assumptions about the properties of the individual distributions (e.g., normality) could be made [25], which we are not willing to do due to lack of prior knowledge.

### Evaluation of the mean response amplitudes of the subpopulations

On the basis of the estimated size of the responsive subpopulation 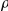 the mean response amplitude of phospho-Erk1/2 within the responsive (r) and the unresponsive (ur) subpopulation can be examined. Therefore, note that the average abundance of phospho-Erk1/2 in the stimulated population is the weighted sum of the average abundances in the individual subpopulations,
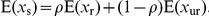
Thereby, 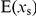 is the mean fluorescence intensity of the stimulated population, 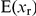 is the mean fluorescence intensity of the responsive subpopulation, and 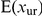 is the mean fluorescence intensity of the unresponsive subpopulation. As 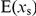 can be measured and 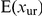 is equivalent to mean fluorescence intensity of the unstimulated control population 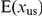, the sole unknown is the average abundance of phospo-Erk1/2 in responsive cells, 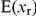. Thus, the equation can be rearranged,
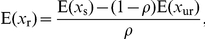
to compute an estimate for the Erk1/2 phosphorylation in the responsive subgroup.

### Evaluation of the cell size specific response

In our study, several cell properties are measured, especially the abundance of phospho-Erk1/2 and the cell size. To analyze the properties of the responsive cells and to perform a biparametric analysis, two-dimensional kernel density estimators are used [22]. Here, the aim was to analyze the property of size of the responsive cells.

At first, the cell size dependency of the percentage of the responsive cells is determined. This is done be computing the two-dimensional kernel density estimates unstimulated and stimulated cell population. These densities are subtracted and the size of the responsive cells is estimated for the individual cell sizes independently. This provides a measure for the cell size dependent responsiveness of the heterogeneous population.

Given the cell size dependent portion of responsive cells, also the size dependency of the response amplitude can be analyzed. Therefore, the analysis introduced in the last subsection is performed for each size interval. This examination allows to answer the question whether a larger phospho-Erk1/2 response is related to a large portion of responsive cells or to the strong response of the responding cells.

### A priori estimate for the required cell number

As outlined above, the quality of the density estimates 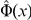, as well as the quality of the estimate of the size of the responsive subpopulation 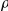, depends on the number of measured cells. To answer the question, how many cells of a heterogeneous cell system have to be analyzed to reliably picture the entire population, an uncertainty analysis is necessary for different amounts of measured cells 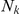.

**Figure 4:**
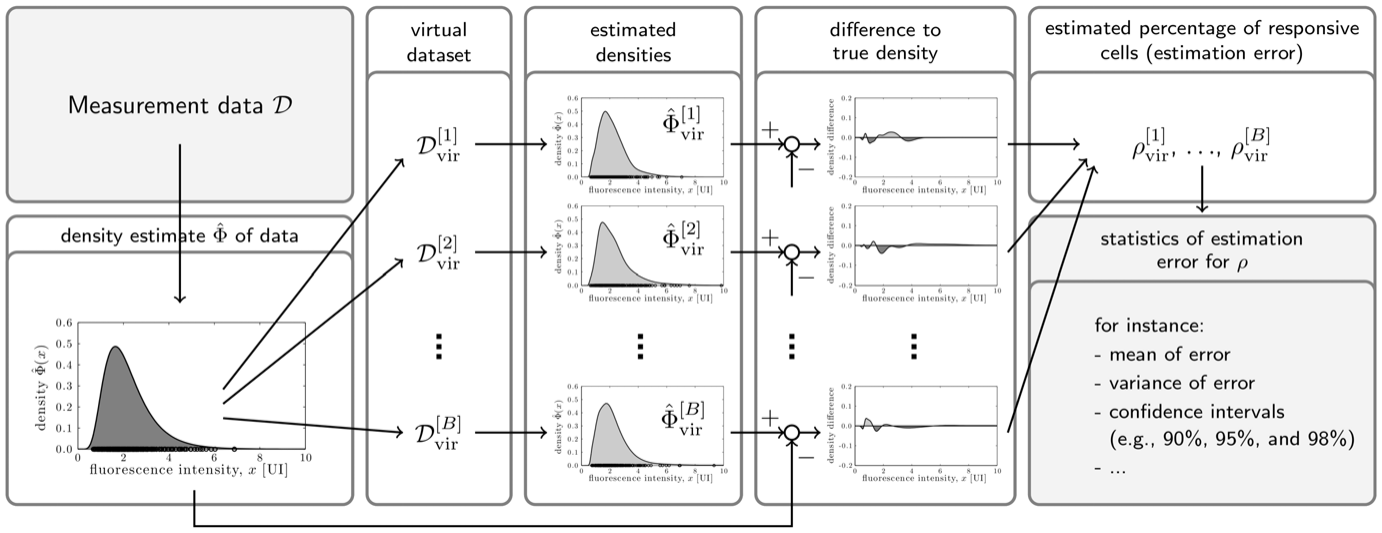
Illustration of parametric bootstrapping procedure employed to determine estimation uncertainties for KDE subtraction method. The individual steps of the uncertainty analysis are: (1) density estimation using measured data, (2) generation of virtual datasets, (3) estimation of population density from virtual datasets, (4) calculation of the difference of the estimated density and the true density, (5) calculation of the classification error, and (6) evaluation of the statistics of obtained estimates.

This uncertainty analysis is performed using parametric bootstrapping. The statistical model 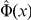 of the cell population is employed to generate a set of virtual measurement data 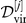, 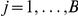, by drawing samples with 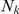 members from 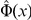. These virtual datasets 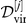, which may be interpreted as a virtual cell culture well, are then employed to determine density estimates 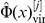. These densities 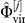 are in turn employed for computing the percentage of responsive cells 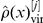. The estimated percentage of responsive cells is computed with respect to 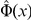. As the estimated densities 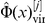 should be identical with the statistical model 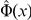, the estimated percentage of responsive cells computed is the estimation error introduced due to the limited cell numbers. Finally, the obtained sample 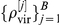 can be examined to obtain insight in the estimation uncertainty. The whole procedure is summarized in Figure 4.

Note, this procedure provides estimates of the uncertainty of 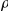 for a certain number of measured cells 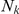. Then being interested in determining the number of cells required to achieve a certain precision, simply the number of cells 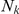 is increased till the requirements are met.

### Test of statistical significance

Throughout the manuscript we used the one-tailed paired t-test for statistical comparison. Pairing is crucial to tackle the problem of inter-individual variability, always present when extracting primary cells from different animals. The variability between animals (here rats) is no measurement noise but still increases the variance of a sample, thus rendering statistical test more conservative. To reduce the effects of inter-individual variability on the outcome of the statistical analysis we used pairing and considered only the relative chance, e.g, of the Erk1/2 activity, in the cells of individual rats. Thus, when testing for statistical significance, instead of verifying that the mean of the sample 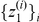 is significant different from the mean of the sample 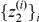, in which 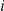 is the index of the individual rat, it has been analyzed whether the mean of the sample 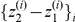 is significant different from zero. This reduced the influence of the inter-individual variability on the outcome of the statistical test, as only measured values stemming from the same rat are compared.

In the whole study p-values
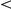
0.05 were considered as statistically significant.

## Results

### KDE subtraction method enables quantification of the portion of NGF responsive DRG neurons

**Figure 5:**
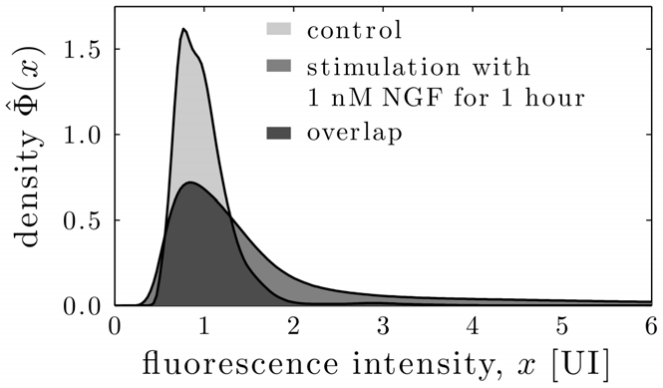
Measured population density for control and stimulation of 1 nM NGF for 1 hour. As there are no two clearly distinct subgroups, the selection of any threshold would result in a non-negligible portion of false positive and false negative cells.

We analyzed the signaling response of DRG neurons to NGF treatment by analyzing the phosphorylation state of the MAP-kinase, Erk1/2, with the newly introduced KDE subtraction method (for details see Materials and Methods). The unstimulated and stimulated populations of these neurons show a large overlap, as depicted in Figure 5. For such populations any given threshold has to result in a large number of false positive or false negative neurons. Thus, thresholding methods fail to provide reliable quantitative information about the sizes of subpopulations [19]. Indeed, when testing wide-spread parametric methods for the analysis of DRG neurons [10], [21], we found that they require a larger separation of the mean fluorescent intensities in the subpopulations for robust result. In addition, they were highly noise sensitive (results not shown). Also, common histogram-based approaches [19], [20] could not be applied as the number of measurable neurons in a normal experiment is to low (there are only about 30.000 neurons in the investigated L1–L6 DRG neurons of a male adult rat).

To overcome these drawbacks, we introduce an approach employing kernel density estimation to analyze subpopulations of DRG neurons responsive to sensitization-stimuli. Given the population density of stimulated and unstimulated cells, the positive part of the difference of both distributions represents the portion of cells, which are surly responding to the stimulus (Figure 3, for a detailed description of the method see Section *Materials and Methods*). Thus, we are able to estimate the percentage of responsive cells.

We stimulated cultures of primary sensory neurons with 1 nM NGF for 60 min. Under these conditions the NGF-induced Erk1/2 phosphorylation level reaches a stable plateau [16]. Using the KDE subtraction method, we estimated that 40
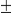
2% of the DRG neurons respond to NGF. This percentage is neither based on any assumption about the intensity distribution in the subpopulation nor depends on investigator-set parameters. Thus, it provides a new quantifiable classification criterion for DRG neurons.

### Reliable size estimates of subgroups from mono-parametric (fluorescence intensity) distributions require 500 measured neurons

The quality of the population density approximation and hence of the calculated percentage of responsive cells is highly dependent on the number and the distribution of the evaluated cells. Nevertheless, an a priori assessment of the number of required measured DRG neurons had to our knowledge never been attempted. One reason might be the lack of large datasets of individual neurons, which became available only recently using QuAM.

**Figure 6:**
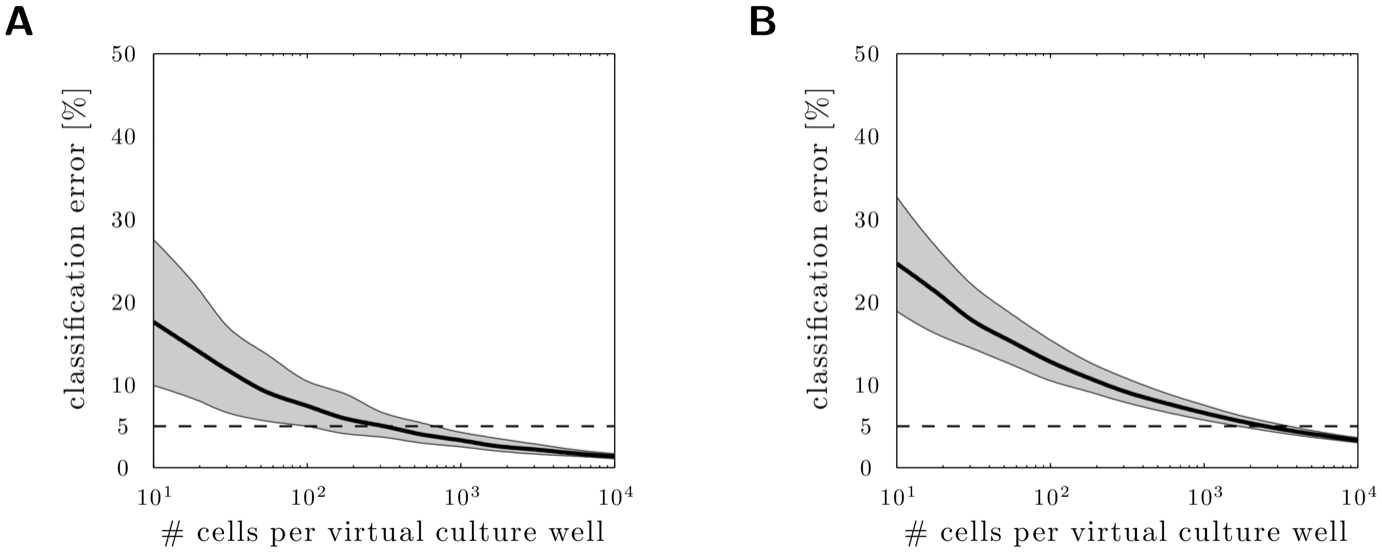
Expected estimation error for percentage of responsive cells depends on the number of measured cells. The estimation error of the percentage of responding cells was estimated for (**A**) one-dimensional datasets (fluorescence intensities) and (**B**) two-dimensional datasets (fluorescence intensities and cell size) of unstimulated cells. The plots show the mean estimation error (black line) as function of the number of measured cells and the 90% confidence interval (gray area). The dashed line represents the acceptable error. For an expected estimation error below 5%, more than 500 cells have to be measured for a mono-parametric KDE-based analysis (**A**) and more than 2,000 cells for a bi-parametric analysis (**B**).

As basis for the evaluation we employed phospho-Erk1/2 intensity data for 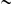
50,000 unstimulated control neurons assumed to reflect the true distribution of phosphorylated Erk1/2. Out of this data pool we sampled randomly virtual culture wells, meaning that the intensity data from these wells have not actually been measured anew but were assembled randomly from the long list of already measured control cells. For these virtual wells the resulting intensity distribution has been computed by kernel density estimation. Comparing the distribution densities of the whole control pool with the distribution densities of each of the sampled virtual wells, we assessed the estimated portion of “responsive cells”. As the virtual wells were derived from the large pool of unstimulated cells and were then compared with the same large pool of unstimulated cells, these computed portion of “responsive cells” represented false positive cells. Therefore, this procedure has established the estimation error in dependence of the chosen size of these virtual wells. The procedure was repeated 1,000 times for each considered virtual culture well size. The resulting classification error is depicted in Figure 6A as a function of the number of measured cells.

Small numbers of analyzed cells reflected the true distribution only partially, thus the estimation error was large. For the numbers of cells we measured in a standard experiment (about 400–600 cells), the expected estimation error was 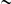
5%, with only small variance (Figure 6A). Obviously, an increased number of measured/analyzed cells led to a reduced estimation error and to a reduced estimation uncertainty. To achieve an expected estimation error below 1% more than 10,000 cells have to be measured.

### The portion of NGF responsive cells is independent of the stimulation time

Given the tools introduced above, novel aspects, such as the response of DRG neurons to NGF stimulation, can be examined in a fully quantitative manner, in awareness of the expected error, and without the need for investigator-set parameters. In the following, we studied dose and time response.

As shown in [26], sensitization in NGF treated animals increases steadily reaching a plateau after 1 hour before starting to fade after about 24 hours. The sensitization increase during the first hour could theoretically be caused by (i) an increase in the percentage of responsive cells, or by (ii) an increase in the response amplitude of the responsive cells.

**Figure 7:**
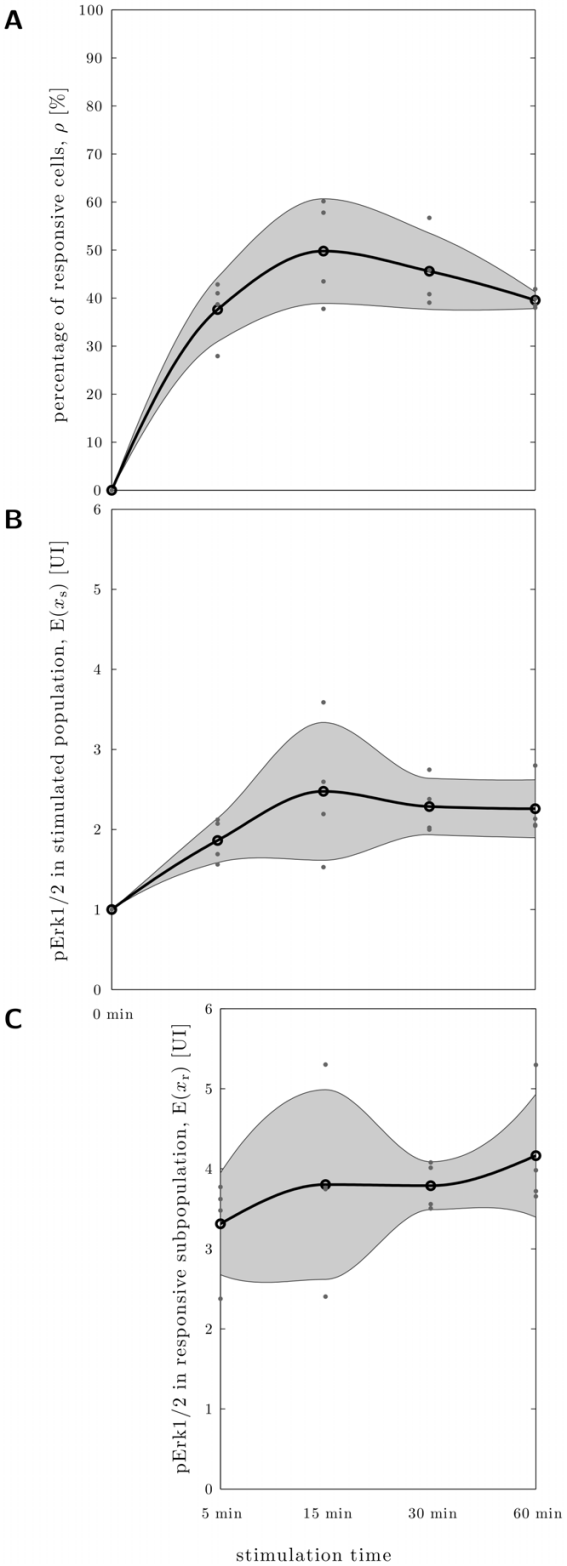
The maximal portion and response amplitude of NGF responsive cells is reached after less than five minutes. **A** Estimated percentages of responsive cells in dependence NGF stimulation time (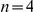 independent experiments with 1,000–4,000 underlying cells per condition). **B** Measured mean phospho-Erk1/2 concentration in the whole NGF-stimulated population in dependence stimulation time. **C** Estimated mean phospho-Erk1/2 concentration in responsive subpopulation, determined from **A** and **B**. The individual data points (dots), their mean (circle) and the one standard deviation confidence interval (gray area) are shown.

To study this, we measured the kinetics of 1 nM NGF-induced Erk1/2 activation after incubation for 5, 15, 30 and 60 min and estimated the percentage of responsive cells. Figure 7A shows that the portion of NGF-responsive cells rose quickly after NGF treatment. Using the one-tailed paired t-test we found for all time points a significant higher portion of responsive cells compared to the cells which were not treated with NGF (Table 1). Between different stimulation lengths, the difference in portion of responsive cells was not significant. Thus, the increase in sensitivity observed in behavioral experiments appears not to be caused by a similar, steady increase of the portion of responsive cells.

**Table 1:** Statistical significance for the dependency of the estimated percentage of responsive cells on stimulation time.

Stimulation time	0 min	5 min	15 min	30 min	60 min
Test vs. 0 min	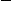	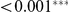	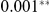	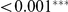	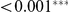
Test vs. 5 min		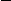	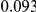	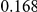	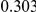
Test vs. 15 min			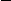	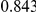	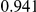
Test vs. 30 min				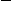	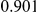
Test vs. 60 min					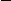

The table provides the p-values obtained by the one-tailed paired t-test. Combinations for which an increase in the stimulation time resulted in a significant increase of the estimated size of the responsive subgroup are marked with one or more asterisks.

### The response amplitude remains constant, independent of the length of NGF stimulation

Next, we measured the time dependence of the mean pErk1/2 intensity of all neurons, exposed to 1 nM NGF (Figure 7B). The pErk1/2 amplitude rose quickly after NGF application. Using the one-tailed paired t-test verified for all time points a significant higher pErk1/2 intensity compared to cells which were not treated with NGF. Again, between different time points there was no significant difference observable (Table 2).

**Table 2:** Statistical significance for the dependency of the estimated mean Erk1/2 activation in responsive cells on stimulation time.

Stimulation time	0 min	5 min	15 min	30 min	60 min
Test vs. 0 min	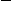	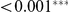	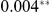	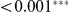	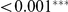
Test vs. 5 min		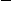	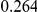	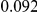	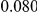
Test vs. 15 min			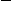	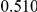	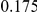
Test vs. 30 min				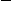	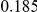
Test vs. 60 min					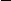

The table provides the p-values obtained by the one-tailed paired t-test. Combinations for which an increase in the stimulation time resulted in a significant increase of the estimated mean Erk1/2 activation in the responsive subgroup are marked with one or more asterisks.

Instead of analyzing the overall population of all responsive and unresponsive neurons, we determined the average response amplitude of the population of responsive neurons (for details see *Materials and Methods*). While we could detect a population of responsive neurons, also a significant increase of Erk1/2 phosphorylation in the responsive subgroup could not be observed as seen in Figure 7C.

### The percentage of NGF responsive cells shows biphasic dose dependence

In behavioral experiments a distinct dose dependence of NGF sensitization has been reported [26]. Therefore, we asked whether the increase of sensitization induced by intradermal injections of increasing NGF concentrations is accompanied by an increase of the percentage of responding cells.

**Figure 8:**
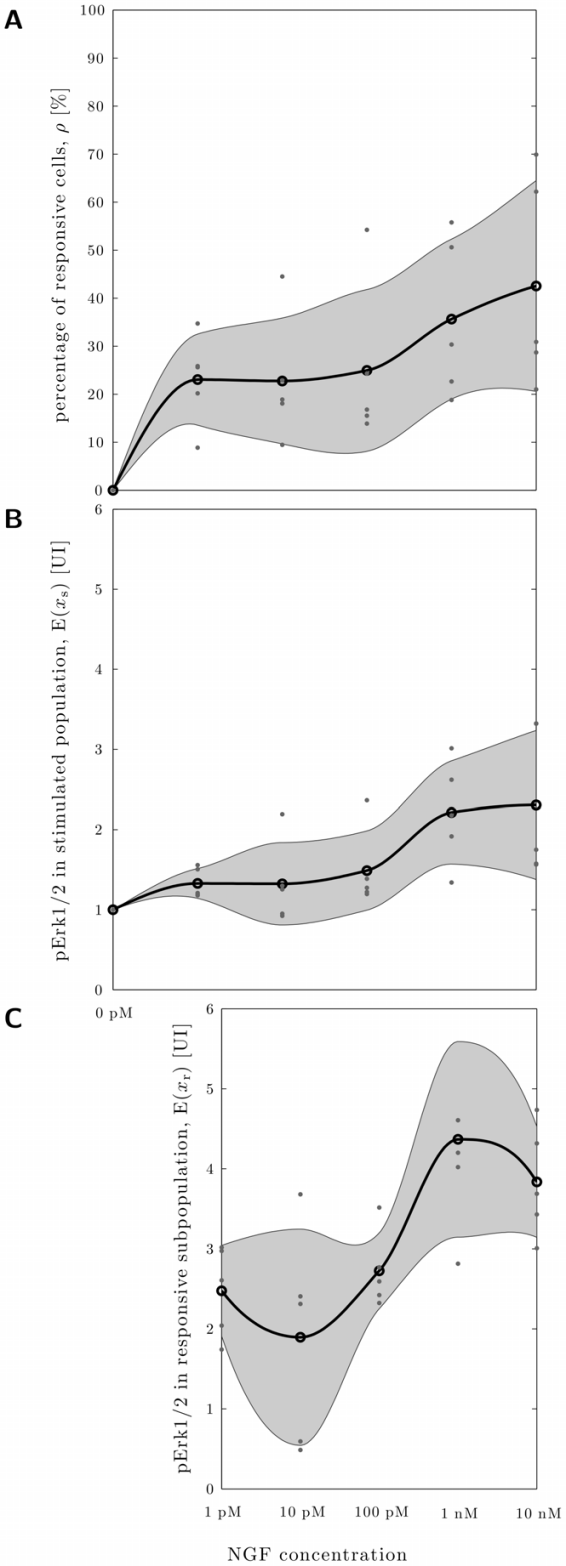
The maximal portion and response amplitude of NGF responsive cells shows biphasic behavior as function of the NGF concentration. **A** Estimated percentages of responsive cells in dependence NGF stimulation time (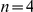 independent experiments with 1,000–4,000 underlying cells per condition). **B** Measured mean phospho-Erk1/2 concentration in the whole stimulated population in dependence NGF stimulation time. **C** Estimated mean phospho-Erk1/2 concentration in responsive subpopulation, determined from **A** and **B**. The individual data points (dots), their mean (circle) and the one standard deviation confidence interval (gray area) are shown.

We recorded a dose response relationship of Erk1/2 phosphorylation after 30 min NGF (1 pM, 10 pM, 100 pM, 1 nM and 10 nM) stimulation and computed for each treatment condition the estimate of the portion of responsive cells. For increasing NGF concentrations the percentage of responsive cells increased (Figure 8A). Interestingly, the dose dependence was neither linear nor did it show a saturation in a common sigmoidal manner with just one saturation plateau.

Statistical analysis employing the one-tailed paired t-test (Table 3) verified that all NFG doses resulted in a significant increase in the number of responding neurons compared to the control. But the dose dependent increase split up into two phases. For NGF concentrations of 1 pM to 100 pM the response of the DRG population was highly similar and statistically indistinguishable, indicating a response plateau. Interestingly, for further increased NFG concentrations of 1 nM to 10 nM a second plateau was reached. This indicated that DRG neurons show a biphasic dose dependence.

**Table 3:** Statistical significance for the dependency of the estimated percentage of the responsive cells on NGF concentrations.

NGF concentration	0 pm	1 pM	10 pM	100 pM	1 nM	10 nM
Test vs. 0 pM	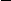	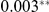	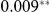	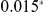	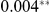	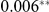
Test vs. 1 pM		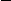	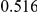	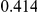	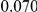	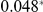
Test vs. 10 pM			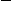	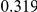	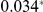	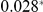
Test vs. 100 pM				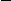	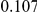	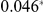
Test vs. 1 nM					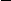	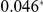
Test vs. 10 nM						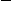

The table provides the p-values obtained by the one-tailed paired t-test. Combinations for which an increase in the NGF dose resulted in a significant increase of the estimated size of the responsive subgroup are marked with one or more asterisks.

### The response amplitude of NGF responsive cells shows biphasic dose dependence

In addition to the portion of responsive cells, we examined whether the increase of sensitization induced by intradermal injections of increasing NGF concentrations is accompanied by an increase of the response amplitude of responding cells. The observed Erk1/2 activation signal strongly increased for 1 and 10 nM NGF. This result was true, based on the analysis of the response amplitude of all neurons exposed to NGF (Figure 8B) as well as by analyzing the responsive neurons (Figure 8C), respectively. The computed levels of statistical significance are provided in Table 4.

**Table 4:** Statistical significance for the dependency of the mean Erk1/2 activation in responsive cells on NGF concentrations.

NGF concentration	0 pm	1 pM	10 pM	100 pM	1 nM	10 nM
Test vs. 0 pM	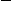	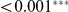	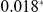	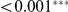	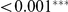	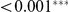
Test vs. 1 pM		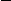	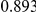	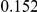	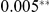	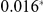
Test vs. 10 pM			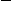	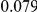	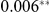	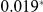
Test vs. 100 pM				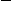	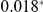	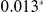
Test vs. 1 nM					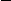	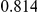
Test vs. 10 nM						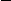

The table provides the p-values obtained by the one-tailed paired t-test. Values marked with Combinations for which an increase in the NGF dose resulted in a significant increase of the estimated mean Erk1/2 activation in the responsive subgroup are marked with one or more asterisks.

### Reliable size estimates of subgroups from bi-parametric (fluorescence intensity and cell size) distributions require 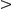
2000 measured neurons

QuAM allows for measuring more than one property of a neuron. This facilitates a more detail subgroup analysis by studying, for example, the size dependency of responsiveness. Before measuring bi-parametric aspects of DRG neurons, we determined in an a priori computational analysis, how many cells have to be measured to ensure a good distribution density estimate for a bi-parametric analysis. We applied the same estimation method as presented before but this time by using a two-dimensional kernel density estimation. Figure 6B depicts the expected estimation error distribution for the two parameters/dimensions, Erk1/2 phosphorylation and cell size. To obtain an estimation error of 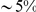 with a low variance, at least 2,000 cells have to be analyzed. Single cell based experiments using such large datasets are still not common in the pain field calling into question many published studies.

### Small-sized neurons respond disproportionally often to NGF

Widely it has been reported, that mostly small neurons respond to noxious stimuli [11]. To our knowledge it has never been investigated, if this reflects only the fact that there are much more small-sized neurons than medium- or large-sized neurons. Alternatively, small-sized neurons could also respond disproportionally often, i.e., in larger numbers in relation to the total number of small neurons.

To test this, we combined the analysis of the degree of Erk1/2 phosphorylation with the analysis of the DRG neuron size. For cells treated with 1 nM NGF for 30 minutes, we analyzed the fluorescence intensity distributions for different cell size intervals as well as the response amplitude in dependence of the cell size. The responsiveness was then calculated as the percentage of responsive cells within a certain size interval divided by the percentage of cells, that fall into this size interval (for details see Section *Materials and Methods*).

**Figure 9:**
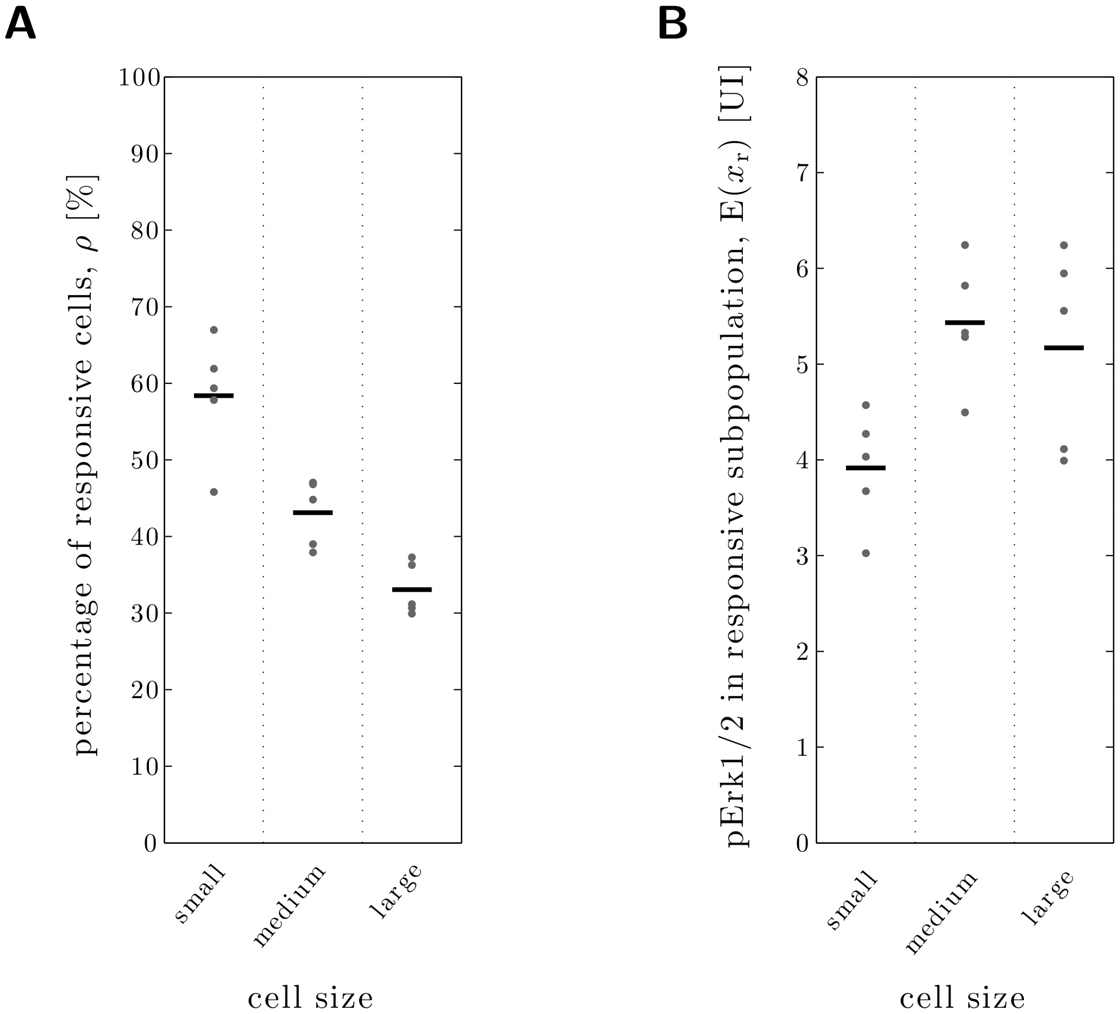
Analysis of size dependency of responsiveness and response strength. **A** Responsiveness observed in individual experiments (dots) and average responsiveness (circle) for small-sized (150–300 m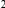), medium-sized (750–900 m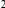), and large-sized (1350–1500 m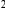) neurons. A statistical analysis using the one-tailed paired t-test verified that the estimated size 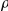 of the responsive subgroup decreased significantly with the cell size (medium vs. small: p-value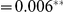; large vs. small: p-value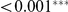; large vs. medium cells: p-value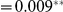). **B** Average pErk1/2 response in the estimated responsive subpopulation (dots) and average over all experiments (circle) for small-sized, medium-sized, and large-sized neurons. Statistical comparison of small-sized neurons with medium- and large-sized neurons using the one-tailed paired t-test indicated that the response of the latter two were stronger (medium vs. small: p-value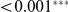; large vs. small: p-value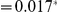). Statistical significant differences between medium-sized and large-sized neurons (p-value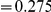) could not be established.

For the analysis we defined three size intervals: small-sized neurons (area of 150–300 µm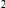; 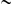
15–20 µm in diameter), medium-sized neurons (area of 750–900 µm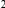; 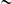
30–34 m in diameter), and large-sized neurons (area of 1350–1500 µm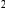; 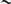
42–44 µm in diameter). For the different size intervals the mean responsiveness was computed (Figure 9A). It is apparent that for larger cell sizes the detected responsiveness is lower. This decrease is statistically significant as confirmed by the one-tailed paired t-test. To our knowledge, this is the first study to analyze DRG neurons on a population basis, which provides evidence that small-sized cells are more frequently responsive than larger ones.

### Medium- and large-sized neurons show the largest response amplitude to NGF

The observed higher response frequency does not indicate if also the response amplitude is higher in small-sized cells. To analyze this, we compared the mean fluorescence intensity of responsive cells of small-, medium-, and large-sized cells. In contrast to the response frequency calculated above, we found, that the fluorescence intensity of small-sized responsive cells was not as high as the fluorescence intensity of medium- and large-sized responsive cells (Figure 9B). This indicated that larger cells respond with stronger Erk1/2 activation. Using again the one-tailed paired t-test we verified that this difference is statistically significant. Therefore, small-sized neurons are more frequently but weaker activated by NGF than larger neurons.

## Discussion

### Quantification of the response of heterogeneous populations using the KDE subtraction method

In this work, we introduced a novel KDE subtraction method for subgroup quantification. This method enables a purely data-based estimation of the portion of responsive cells, without knowledge of a threshold or a distribution shape. Our approach merely requires single-cell population data of stimulated and control cells, here obtained by quantitative microscopy, and calculates the percentage of responsive cells from the difference between the measured population densities.

The proposed KDE subtraction method outperforms other methods available in the literature such as ratio analysis-, mixture model-, or histogram-based approaches for the considered dataset of DRG neurons. Ratio analysis-based approaches [21] become extremely noise sensitive if the left tail of the stimulated and unstimulated population are highly similar. This is the case for most measurements of DRG neurons as only some of the neurons show a strong response and many if not most remain unresponsive. In the limit of identical tails, the results of ratio analysis-based approaches then only depends on the measurement noise and are thus inherently inaccurate [21].

The mixture model [10], [27] on the other hand does not suffer this extreme sensitivity. Nevertheless, the results depend strongly on the parameterization of the density of the subpopulations. In particular, if the subpopulations overlap it is difficult to determine reliable parameter estimates.

Finally, the (modified) histogram subtraction methods [19], [20] commonly in use, e.g., in flow cytometry, require large datasets of often more than 10,000 cells per individual experiment due to the inefficient density estimation of histograms [22], [27]. In addition, the required number of data strongly increases with the number of considered features, which is why histograms are in general not used to study multi-dimensional datasets [22]. As in general only few tens of thousands of neurons can be recovered from a single rat, the excessively large datasets required by histogram-based methods prohibited the use of this method for the analysis of primary cells like DRG neurons.

Accordingly, the KDE subtraction method is an urgently needed tool for the analysis of largely overlapping heterogenous populations of medium-sized datasets. Nicely, if the data show a clear threshold (Figure 1A), e.g., due to a strong response of the responsive subgroup, the result of the KDE-based analysis converges to the result of thresholding methods. Therefore, we believe that the KDE-based approach is the method of choice for quantitative analysis of heterogeneous populations such as stimulus responsive subgroups of DRG neurons.

### Data-driven experimental planning using bootstrapping methods

Beyond an improved analysis of experimental data, also more sophisticated methods for experimental planning are desirable. During recent years, model-based experimental planning became more and more common in experimental studies as a tool to reduce the overall number of required experiments [28]–[32]. Unfortunately, in many biological studies mathematical models of the processes are not yet available. Therefore, we propose a procedure for experimental planning, which does not rely on a process model.

We suggest a data-driven approach to determine the number of single-cell measurements necessary for a reliable population estimate. Our approach employs a non-parametric model of the data, derived by kernel density estimation (see for details [24]). Given this data model, bootstrapping methods enable the assessment of expected uncertainties of the quantity of interest – in this study the size or the amplitude of the responsive subpopulation, respectively – before the actual experiment is performed. To achieve this a simulation of the planned experiments with varying data sizes is performed using the available pool of data. This allows the prediction of the expected information content and the uncertainties of the experiment, given by the available data. If the information content is not satisfactory the experiment may be adapted, e.g., the number of measured cell may be increased.

To the best of our knowledge, such data-driven approach to experimental planning has so far not been used in the context of nociceptive neuron subpopulation analysis. Merely, by [16] a first analysis was attempted to determine the number of cells needed to infer information about the mean signaling status in such a population. The method we propose is more general and might be applied independent of the tool used for data analysis.

Using this bootstrapping approach, we could show that a bi-parametric analysis requires for our system approximately four times more cells than a mono-parametric analysis. Accordingly, the experimental setup was adapted to allow for an in-depth study of the NGF response of DRG neurons.

### Reported DRG neuron subgroup sizes are highly variable and unreliable

DRG neurons are frequently used to investigate cellular mechanism underlying pain and pain sensitization. One aspect of considerable interest is the identification and quantification of sensory neuron subgroups [33], [34]. Literature data on sizes of DRG subpopulation are commonly based on counting positive and negative cells as judged by a trained investigator. Thus, this kind of subgroup classification is based on thresholding [35]. But thresholding can be problematic as it becomes apparent by comparing reports about population sizes of DRG-subgroups. For example the IB4 binding subgroup is described to contain 40–70% [36]–[38], the TRPV1 expressing subgroup 30–80% [17], [18], the NGF receptor expressing subgroup 35–70% [37], [39], and the NGF responsive subgroup 30–60% [16], [35] of all DRG neurons.

These differences could first of all reflect the application of different thresholds. But our quantitative analysis of several aspects of nociceptive neurons, such as protein expression and signaling activation state, indicates also a more fundamental problem. The distributions of the investigated cellular properties are broad and overlap strongly with the distributions of the respective negative controls. This suggests that the concept of clearly separated subgroups – underlying most of the published studies – is an oversimplification. Thereby, thresholding causes quantification errors in the analysis of DRG neuron subgroups, explaining the discrepancies between studies. We believe, that these errors and discrepancies can be reduced by more sophisticated data analysis procedures, such as the proposed KDE subtraction method.

### Investigation of dynamic signaling components increases antibody specificity and sensitivity of antibody-based assays

A further source of variability/uncertainty in the determination of subgroups and subgroup sizes are the antibody-derived signals. Antibody-based classification of subgroups is limited by the properties of the respective antibody, in particular its specificity and sensitivity. Antibody-derived signals summarize specific, cross-reactive and unspecific interactions with cellular components. Specific signals result from high affinity binding of the antibodies to the protein of interest, which is mediated by an interaction of the antigen binding sites and its target epitope. In addition, sometimes the antigen binding sites also bind to highly similar epitopes, a phenomenon called cross-reactivity. Finally, parts other than the antigene binding side might interact with charged areas present on various molecules. Such interactions have usually lower affinities and are termed unspecific binding. While the specific binding is the signal of interest, cross-reactive and unspecific binding cause an undesired “background” signal.

To tell apart specific binding and unspecific/cross-reactive binding, mainly knock-out animals, tissues, and cells have been employed, which lack the epitope/protein of interest. While widely accepted, this approach is often not feasible, e.g., if animals are studied for which knock-outs are not routinely performed, such as rats.

In our study, we avoid the analysis of epitopes which are permanently present. Instead, we analyze changes in epitope abundances, such as the differential Erk1/2 phosphorylation. Our focus on dynamic changes in signaling components avoids much of the challenging identification of unspecific and cross-reactive binding. As levels of unspecific and cross-reactive signals are highly similar in stimulated and unstimulated cultures, the rise (decline) of the detected signal after stimulation must be caused mostly by an increase (decrease) of specifically binding antibodies. Thus, this change in detected signal reflects the varied epitope availability. Hence, measuring a dynamic alteration of a component, such as the degree of phosphorylation of the Erk1/2 in response to, e.g., NGF stimulation, and calculating the difference between these two conditions eliminates much if not all of the unspecific or cross-reactive signal. This greatly increases the degree of specificity.

Secondly, the focus on signaling components results in an improved sensitivity. Transmembrane proteins, such as receptors, and/or ion channels, are usually expressed only in small quantities. Furthermore, they often share common motives and/or are heavily and dynamically post-translationally modified by, e.g., glycosylation, rendering it difficult to produce high quality antibodies against them. Investigation of the dynamics of signaling components downstream of receptors allows to overcome some of these problems as their abundance is commonly large. Thus, both aspects, increased specificity as well as increase sensitivity, can result in better signal-to-noise ratios.

### Analysis via the KDE subtraction method allows for quantification of signaling-dynamic based DRG subgroups

Much effort has been invested to identify extracellular mediators as well as effector structures such as ion channels involved in sensitization. We believe that the intracellular machinery [14], which integrates the various sensitization inputs, computes the response of the neuron, and regulates not only one but a multitude of effector proteins, still requires extensive research. Tools for the investigation of endogenous signaling components in heterogeneous primary neurons so far hardly allowed for an in-depth analysis of the intracellular sensitization machinery. Using automated microscopic methods and the here proposed KDE subtraction method enables the quantitative investigation of, e.g., phosphorylation states of endogenous signaling components as surrogate measurements of their activity.

We applied the KDE subtraction analysis approach to investigate the subgroup of NGF responsive DRG neurons. We found 40
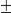
2% of the neurons to respond after 60 minutes to 1 nM NGF with an increase in phosphorylation of Erk1/2. As our method of quantification provides a conservative measure, we can disregard the reported smaller population sizes for NGF-responsive neurons. In addition, while indeed some more neurons might express NGF-receptors, in the end it is most important for the investigation of sensitization mechanisms to identify the neurons, which are actually really activated/sensitized by NGF treatment as visible by the increased phospho-Erk1/2 levels.

### Switch-like activation of Erk1/2 after NGF treatment

Shifting the research focus away from static subgroup markers as ion channel expression to dynamic ones as signaling components inspires also the investigation of dynamic aspects such as kinetics. While dependence of cellular functionality on kinetic and dose response aspects of Erk1/2 activation have frequently been described in secondary cell lines [40]–[45], also subgroup functionality may depend rather on the dynamics and kinetics of an activation event than on the activation itself. The KDE subtraction method opens the door to analyze such population specific events.

Analyzing fundamental dynamic response aspects such as the amplitude and the proportion of responding neurons over time yields surprising results. Our data suggest, that the response amplitude is time independent (Figure 7C). Especially with a multicomponent signaling system such as MAPK/Erk1/2 cascade one might expect that the amount of active Erk1/2 slowly increases over time. Our findings apparently indicate, that the amplification by the preceding kinases, each of which activates multiple substrate kinases, which in turn activate multiple Erk1/2 kinases, is so fast that the phenotypic result is rather a switch-like behavior of Erk1/2 activation.

Interestingly, the sensitization in the animal establishes itself over about 30 min [26]. A potential mechanism underlying this increase in sensitivity could therefore be the activation of increasing numbers of nociceptive neurons. But also this appears not to be the case as we find the portion of responding neurons to be time independent (Figure 7A). This suggests that the step determining the behavioral sensitization-kinetic is not the signaling cascade up to Erk1/2 phosphorylation but the downstream events.

### Biphasic dose response indicates different NGF activated subgroups

Besides the NGF-induced Erk1/2 activation kinetics, the dose response behavior has been analyzed. The analysis showed a biphasic response indicating the existence of at least two distinct subgroups which are defined by their signaling state. The first phase of the dose response (plateau between 1 pM and 100 pM NGF) pictures the first subgroup. At least 23% (Figure 8A) of the DRG neurons belong to this subgroup responding already to low doses of NGF and showing a low level of Erk1/2 phosphorylation (factor two compared to the unresponsive cells (Figure 8C)). The second subgroup responds only to higher NGF concentrations (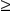
1 nM) and explains the second rise in the dose response plot (Figure 8A). We provided evidence that this subgroup exhibits a significant increase of Erk1/2 activation (Figure 8C, Tab. 4). Unfortunately, our methods cannot discriminate whether these subgroups are overlapping or non-overlapping.

The subgroup specific responses might be due to variations in the expression of the NGF receptor TrkA. Alternatively, the abundance of p75, which can bind to TrkA and enhance its NGF affinity [46], or an altered regulation of Erk1/2 activity further downstream may cause the observed subgroup differences. So far, we cannot distinguish between these alternatives. Co-staining of phospho-Erk1/2 and TrkA or p75 might solve the molecular underpinning of this phenomenon, whenever suitable antibodies become available. Nevertheless, the identification of the existence of three differently responsive subgroups (unresponsive, weakly responsive, and strongly responsive cells) provides a novel insight into functional differences of nociceptive signaling in DRG neurons.

As no thresholds are assumed, complex phenomena based on graded response differences can be unraveled. This is important, as complex and graded subpopulation structures are also observed for other DRG markers like IB4 binding [47]. The KDE-based approach allows to quantify these specific subgroups and opens the door to further in depth analysis.

### KDE subtraction method identifies individual responses of DRG subgroups characterized by cell size

Defining the responsive subgroup not only allows to investigate kinetic and dose dependent activation behaviors, but also to investigate characteristics of the responsive subgroups in respect to expression of protein markers or cell size. In this study, we analyzed the size properties of the responsive subgroups. We found that small-sized neurons (150–300 µm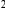) are more responsive than larger neurons, as suggested but not proven by a large number of publications (Figure 9A). But in contrast and so far not described, we found medium-sized cells (750–900 µm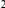) to respond strongest (Figure 9B). Which aspect contributes more to pain sensitization, number or amplitude of responding neurons, is not known.

Analyzing nociceptive neurons on the basis of signaling kinetics is new. As indicated in the subsequent chain of arguments, this might give a new aspect helping to identify, for instance, the subgroup essential for mechanical sensitization. Cells positive for the marker IB4 have been described to be in average larger than neurons positive for the classical markers of C-fibers, TRPV1 and CGRP [48]. We find NGF to activate Erk1/2 in small- but also medium- and large-sized IB4-positive neurons. As IB4-positive neurons are proposed to mediate mechanical pain but not heat pain [34], these medium- and large-sized neurons characterized by higher NGF responsiveness might be the IB4-positive subgroup involved in NGF-mediated mechanical hyperalgesia. Corroborating electrophysiological studies are necessary but are currently beyond the scope of realistic investigation as they require for the quantitative analysis of an electrophysiological data based population study a very large number of recorded neurons.

### Conclusion

To circumvent the shortcomings of thresholding methods, we introduced the KDE subtraction method to quantify the responsive cells. This analysis tool closes the gap between thresholding methods and the highly quantitative analysis tools like histogram subtraction which require in general 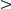
10,000 measured cells per individual experiment and are therefore not feasible when studying primary sensory neurons. By combining the KDE subtraction method with QuAM, which can greatly increase the numbers of investigated and quantified cells, we rendered a fully quantitative analysis of DRG neurons feasible.

We quantified and characterized the NGF responsive subgroups and found DRG-subgroup specific differences in dose dependent NGF responsiveness as well as in the magnitude of NGF-induced Erk1/2 signaling. This indicates a subgroup specific signaling regulation. Our approach renders an in-depth analysis of signaling activation in heterogeneous nociceptive neurons manageable, which goes beyond “yes or no” evaluations. Similar to the study of the size dependence of the response, the proposed analysis of Erk1/2 activation can also be combined with common nociceptive markers, e.g., IB4 binding, CGRP and TRPV1 expression. As intracellular signaling is a promising therapeutically target in pain, such knowledge of signaling patterns is the beginning of the search for subgroup specific signaling targets.
